# Robot-assisted minimally invasive esophagectomy versus video-assisted thoracoscopic esophagectomy versus open esophagectomy for locally advanced esophageal cancer after neoadjuvant therapy: a systematic review and network meta-analysis

**DOI:** 10.3389/fonc.2025.1631672

**Published:** 2025-11-20

**Authors:** Yufei Zhou, Qiangqiang Zheng, Yunfeng Zhou, Xiong Liu, Wei Chen, Yusong Lu, Yang Yuan

**Affiliations:** 1 Department of Cardiovascular Medicine, Chengdu Integrated Traditional Chinese Medicine (TCM) & Western Medicine Hospital, Chengdu, China; 2 Department of Thoracic Surgery, West China School of Public Health and West China Fourth Hospital, Sichuan University, Chengdu, China

**Keywords:** esophageal cancer, neoadjuvant therapy, esophagectomy, minimally invasive surgery, robot-assisted surgery, network meta-analysis

## Abstract

**Background:**

Esophageal cancer (EC) remains a lethal malignancy with poor survival outcomes despite multimodal therapy. While minimally invasive techniques like video-assisted thoracoscopic esophagectomy (VATE) and robot-assisted minimally invasive esophagectomy (RAMIE) have gained traction over open esophagectomy (OE), their comparative safety, efficacy, and survival benefits in patients receiving neoadjuvant therapy remain underexplored.

**Methods:**

We conducted a Bayesian network meta-analysis on data from seven studies (n=1847 patients) to compare OE, VATE, and RAMIE after neoadjuvant therapy for locally advanced EC. Outcomes included complication rates, operative time, R0 resection, lymph node yield, and 3-year overall survival (OS).

**Results:**

No significant differences were observed in R0 resection rates (RAMIE vs. OE: OR = 1.03, 95% CI 0.25–4.70; VATE vs. OE: OR = 1.37, 0.67–3.45), lymph node dissection (RAMIE vs. OE: WMD = 1.56, −3.29–6.43; VATE vs. OE: WMD = 1.05, −2.24–4.53), or 3-year OS (VATE vs. OE: HR = 1.14, 0.70–1.85). RAMIE ranked highest for reducing complications (SUCRA = 52.5%), while OE showed shorter operative time (SUCRA = 94.0%). Achieving R0 resection ranking: RAE (SUCRA 47.3%), OE (SUCRA 43.8%), and VATE (SUCRA 8.9%). In lymph node dissection, OE had the highest probability of being superior (59.5%), markedly outperforming RAMIE (21.3%) and VATE (19.2%). Survival outcomes were comparable across all approaches.

**Conclusions:**

OE, VATE, and RAMIE demonstrate equivalent oncological efficacy in EC after neoadjuvant therapy. Perioperative advantages differ: RAE may lower complications, whereas OE offers procedural efficiency. Surgical selection should prioritize individualized risk-benefit assessment, anatomical considerations, and institutional expertise. Prospective trials are warranted to validate these findings and refine technique-specific indications.

## Introduction

Esophageal cancer (EC) represents a highly lethal malignancy with considerable global incidence and mortality, as evidenced by the 604,000 new cases and 544,000 deaths reported in 2020, positioning it as the seventh most prevalent and sixth most fatal cancer worldwide (1). EC is classified into two pathological subtypes: esophageal squamous cell carcinoma (ESCC) and esophageal adenocarcinoma (EAC). In China, ESCC predominates, accounting for approximately 90% of esophageal cancer cases (2), whereas in Europe and America, 70–80% of esophageal cancers are adenocarcinomas (3). The prognosis for EC remains poor, with an 5-year overall survival (OS) rate of less than 30%, and post-surgical survival outcomes are similarly unsatisfactory (4, 5). Recently, neoadjuvant therapies, such as neoadjuvant chemoradiotherapy (NCRT) or neoadjuvant chemotherapy (NCT), followed by surgical intervention, have emerged as the standard treatment approach for patients with locally advanced EC (6). High-quality clinical studies, including ESCORT-NEO, have demonstrated that the combination of neoadjuvant chemotherapy and immunotherapy (NCIT) is safe and efficacious for patients with locally advanced EC (7). We can discuss the case in a multidisciplinary commission with the nutritionist and the oncologist for neoadjuvant treatment (8). Following neoadjuvant therapy, numerous patients with EC exhibit tumor regression. However, surgical intervention becomes increasingly complex and hazardous due to edema, adhesion, and scar tissue formation within the esophageal bed. Additionally, some patients may experience adverse events related to the treatment, resulting in compromised physical health (9). Investigating surgical approaches that prioritize patient safety, efficacy, and long-term survival outcomes is imperative.

The progression of minimally invasive surgical techniques has facilitated a shift in EC surgery from the conventional open esophagectomy (OE) to minimally invasive esophagectomy (MIE) (10). MIE is a surgical approach for esophageal cancer that primarily utilizes video-assisted thoracoscopic esophagectomy (VATE). MIE includes procedures such as video-assisted thoracoscopic esophagectomy (VATE) and total minimally invasive esophagectomy (TMIE). Among these techniques, VATE is considered the foundational technology and cornerstone of MIE within the thoracic field, and it is extensively utilized in clinical practice. In this study, We concentrates on VATE, include hybrid esophagectomy procedures. The two main procedural classifications under this paradigm are the McKeown esophagectomy (three-field approach) and the Ivor-Lewis esophagectomy (two-field approach). Concurrently, the global adoption of robot-assisted minimally invasive esophagectomy (RAMIE) is rising (11). A retrospective study compared OE and VATE for EC. The findings indicated that VATE significantly reduced operation time and postoperative hospital stay, while also enhancing the 5-year OS rate of patients (12). A retrospective study compared RAMIE with OE, revealing that the former is associated with a reduced length of hospital stay and a lower reoperation rate. However, it also showed an increased incidence of postoperative pulmonary embolism, with no significant difference in long-term survival between the two methods (13). Additionally, clinical trials have compared RAMIE with VATE. These trials indicated that RAMIE may enhance lymph node dissection in patients with esophageal cancer. However, no significant differences were observed in mortality, complication rates, or perioperative outcomes between the two groups (14). While several studies have examined the advantages and disadvantages of various surgical techniques, there is a paucity of clinical research specifically addressing surgical methods for EC patients who have undergone neoadjuvant therapy, with existing studies often constrained by limited sample sizes. Consequently, it is imperative to perform a network meta-analysis utilizing all available data to comprehensively investigate the safety, efficacy, and long-term survival outcomes associated with different surgical approaches-namely, OE, VATE, and RAMIE-in the context of locally advanced EC following neoadjuvant therapy.

## Methods

### Protocol and registration

We registered our protocol on the PROSPERO (CRD42024620991). The work has been reported in line with Preferred Reporting Items for Systematic Reviews and Meta-Analyses (PRISMA) and assessing the methodological quality of systematic reviews (AMSTAR) Guidelines (15, 16).

### Search strategy

We conducted a systematic literature search of PubMed, EMBASE, and the Cochrane Library from inception to November 30, 2024. The utilized search terms were: (Esophageal cancer OR Esophageal Neoplasm OR gastroesophageal junction carcinoma) AND (Neoadjuvant Therapy OR Neoadjuvant Chemotherapy OR Neoadjuvant Chemoradiotherapy OR Neoadjuvant chemo-immunotherapy) AND ((Surgery OR Surgery, General) AND (Randomized controlled trial OR Cohort studies OR Case-control study).

### Study selection

Two reviewers (Yang Yuan and Qiangqiang Zheng) independently reviewed the available literature. A third author (Yufei Zhou) resolved any differences in their assessments. We included the studies which met the predefined eligibility criteria.

### Inclusion criteria

The inclusion criteria were as follows (1): Patients with pathologically confirmed locally advanced EC were included in the study (2), All participants underwent neoadjuvant therapy prior to surgical intervention, followed by OE, RAE, or VATE (3), Provision of the safety and efficacy, as well as long-term survival outcomes, with associated 95% confidence intervals (CIs), and (4) The analysis incorporated data from case-controlled studies, cohort studies, and randomized controlled trials (RCTs).

### Exclusion criteria

The exclusion criteria were as follows (1): Patients with different types of tumors (2), The necessary outcomes were not revealed, making it impossible to compute them from the initially published information (3), Lack of sufficient data, and (4) Repeated studies.

### Literature quality evaluation

The quality assessment criteria comprised three domains: Subject selection (4 items: (1) representativeness of exposed cohort, (2) selection of non-exposed cohort, (3) ascertainment of exposure, and (4) demonstration that outcome was not present at study initiation) with maximum 1 point per item, Comparability (1 item: (5)control for confounding factors in design/analysis) with maximum 2 points, Outcome assessment (3 items: (6) outcome evaluation methods, (7) adequacy of follow-up duration, and (8) completeness of follow-up for both cohorts) with maximum 1 point per item.

### Statistical analysis

#### Network meta-analysis

For the outcomes of overall complication rate, operative time, R0 resection rate, and number of lymph nodes retrieved, we performed a Bayesian network meta-analysis with the gemtc package in R software (version 4.3.1). We visualized the ranking probabilities of different interventions through rankogram plots. We assessed the consistency between direct and indirect evidence within the network by the node-splitting model, P < 0.05 defined as statistical significance. We evaluated the Convergence of the Markov chain Monte Carlo (MCMC) simulations by the potential scale reduction factor (PSRF), where values approaching 1.0 indicated satisfactory convergence (17).

#### Pairwise meta-analysis

For the outcome of 3-year OS, pairwise meta-analyses were conducted with the meta package (version 4.3.1) in R. We reported Hazard ratios (HRs) with 95% confidence intervals (CIs) as pooled estimates. Heterogeneity across studies was quantified using the I² statistic and Cochran’s Q-test P-value. A fixed-effects model was applied when I² < 50% or P > 0.1; otherwise, a random-effects model was employed (18). Statistical significance was defined as a two-sided P < 0.05.

### Sensitive analysis

Of the seven studies included in the analysis, only one was a RCT, whereas the other six were cohort studies. Given the potential for confounding bias due to the heterogeneity of study designs within the meta-analysis, a sensitivity analysis was conducted by excluding the sole RCT to assess the robustness of the aggregated findings.

## Results

### Overview of literature search

**Figure 1:**
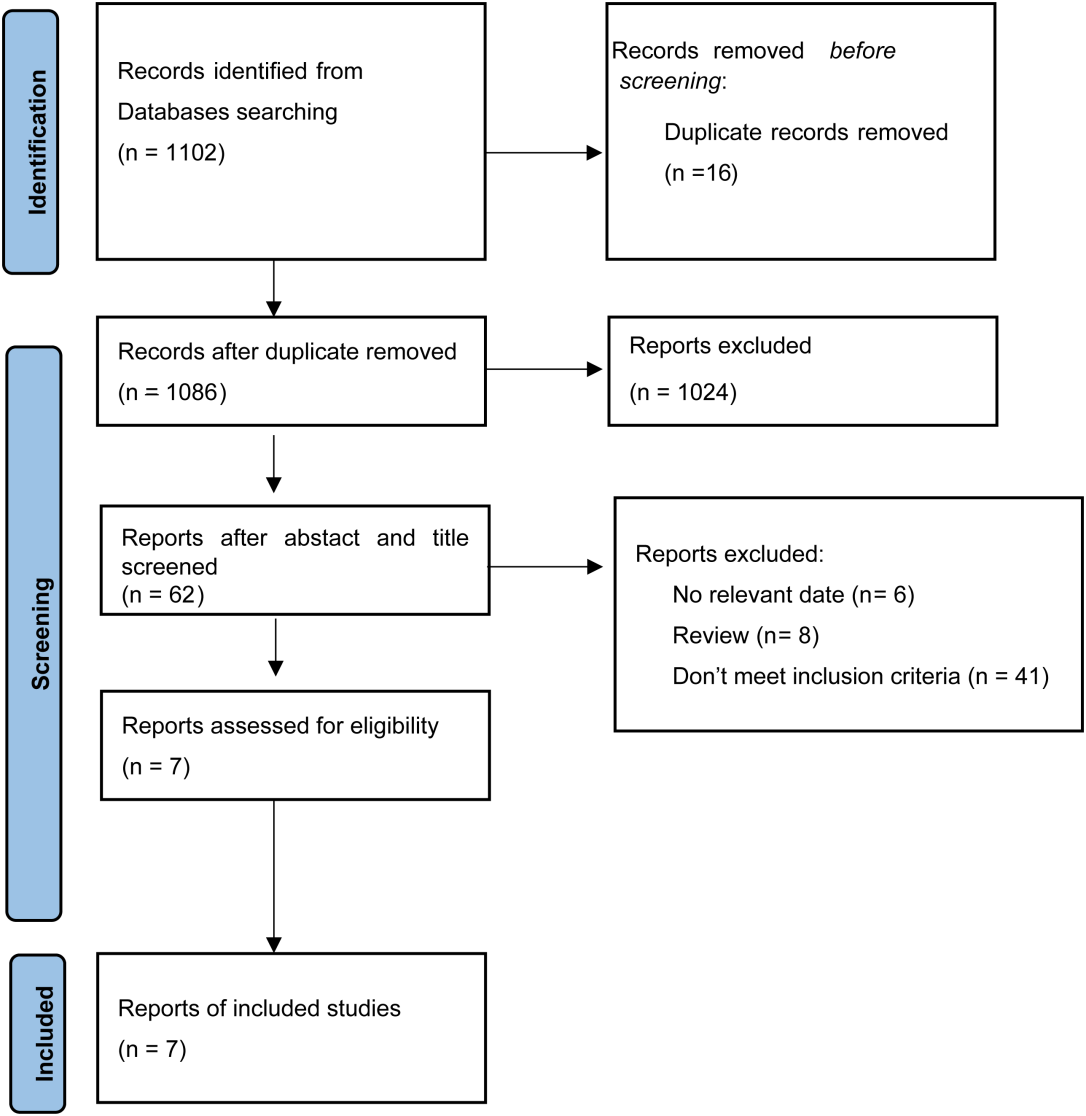
Study flow diagram of study selection process.

**Figure 1** summarizes the flowchart of study selection. Computer-based database searches and complementary manual search retrieved a total of 1,102 relevant articles. Articles were exported to Endnote 20, after removing 16 duplicates, we read the titles and abstracts of the 1,086 studies left, 1,024 studies were excluded because they were letters, meta-analyses, meeting abstracts, videos, editorials, comments and case reports. After meticulously reading, 55 studies were excluded because 6 studies had no relevant data, 8 studies were reviews, and 41 studies did not meet inclusion criteria. In total, 7 eligible studies were enrolled in this NMA.

### Characteristics of studies included in the network meta-analysis

Seven articles published between 2017 and 2024, consisting of a total number of 1,847 participants were included in this NMA. There were six two-arm studies and one three-arm studies with four comparisons, including RAS, OE, and TE. Briefly, study sample sizes ranged from 28 to 1,059. Among all individuals, 79 were treated with RAMIE, 730 with OE, and remaining 1,038 were treated with VATE. The detailed characteristics of the included studies were displayed in **Table 1**.

**Table 1:** Basic characteristics of the included studies.

Study	Country	Design	Total sample size	Intervention	Sample size	Gender (M/F)	Age, years	Median follow-up time, months	Type of anastomosis	Location of anastomosis	Extent of lymphadenectomy
Jae, 2019 (19)	Korea	Retrospective	146	RAMIE	35	30/5	61.2 ± 6.4	21.2	Stapled	Cervical or intrathoracic	Standard
				OE	111	102/9	61.6 ± 7.8	26.5	Stapled	Cervical or intrathoracic	Standard
Jennifer, 2017 (20)	Europe	RCT	115	VATE	59	43/16	61.8 ± 8.4	27	Not described	Not described	Not described
				OE	56	46/10	62.3 ± 8.4	22	Not described	Not described	Not described
Fan, 2022 (21)	China	Retrospective	139	VATE	96	66/30	/	11.6	Not described	Not described	Standard
				OE	43	36/7	/	11.6	Stapled	intrathoracic	Standard
Keijiro, 2023 (22)	Japan	Prospective	262	VATE	131	109/22	67 (46–79)	49.5	Not described	Cervical or intrathoracic	Standard
				OE	131	113/18	66 (37–80)	49.5	Not described	Cervical or intrathoracic	Standard
Sercan, 2024 (23)	Turkey	Retrospective	28	RAMIE	13	6/7	64.7 ± 8.4	/	Stapled	Cervical or intrathoracic	Standard
				VATE	15	5/10	59.0 ± 7.8	/	Stapled	Cervical or intrathoracic	Standard
Wu, 2024 (24)	China	Retrospective	98	RAMIE	31	29/2	62.9 ± 9.3	3	Stapled	Cervical or intrathoracic	Standard
				VATE	31	29/2	61.8 ± 8.3	3	Stapled	Cervical or intrathoracic	Standard
				OE	36	32/4	65.0 ± 7.4	3	Stapled	Cervical or intrathoracic	Standard
Chen, 2022 (24)	China Taiwan	Retrospective	1059	VATE	706	677/29	53.94 ± 8.28	36	Not described	Not described	Not described
				OE	353	342/11	54.51 ± 8.39	36	Not described	Not described	Not described

### Quality of evidence

This systematic review included six cohort studies, with methodological quality assessments rating four as moderate and two as high quality. Additionally, the incorporated randomized controlled trials demonstrated low risk of bias according to standardized evaluation criteria (**Table 2** and **Table 3**).

**Table 2:** Results of quality evaluation of cohort study (scores).

Cohort study	Selection	Comparability	Outcome	Quality scores
Jae	4	2	1	7
Fan	4	2	0	6
Keijiro	4	3	2	9
Sercan	4	2	0	6
Wu	4	2	1	7
Chen	4	3	2	9

**Table 3:** Results of quality evaluation of RCT.

RCT	Selection bias		Reporting bias	Performance bias	Detection bias	Attrition bias	Other bias
	Random sequence generation	Allocation concealment	Selective reporting	Blinding of participants and personnel	Blinding of outcome assessment	Incomplete outcome data	
Jennifer	Low risk of bias	Low risk of bias	Low risk of bias	Low risk of bias	Low risk of bias	Low risk of bias	Low risk of bias

### Network meta-analysis

#### Overall complication rates

**Figure 2:**
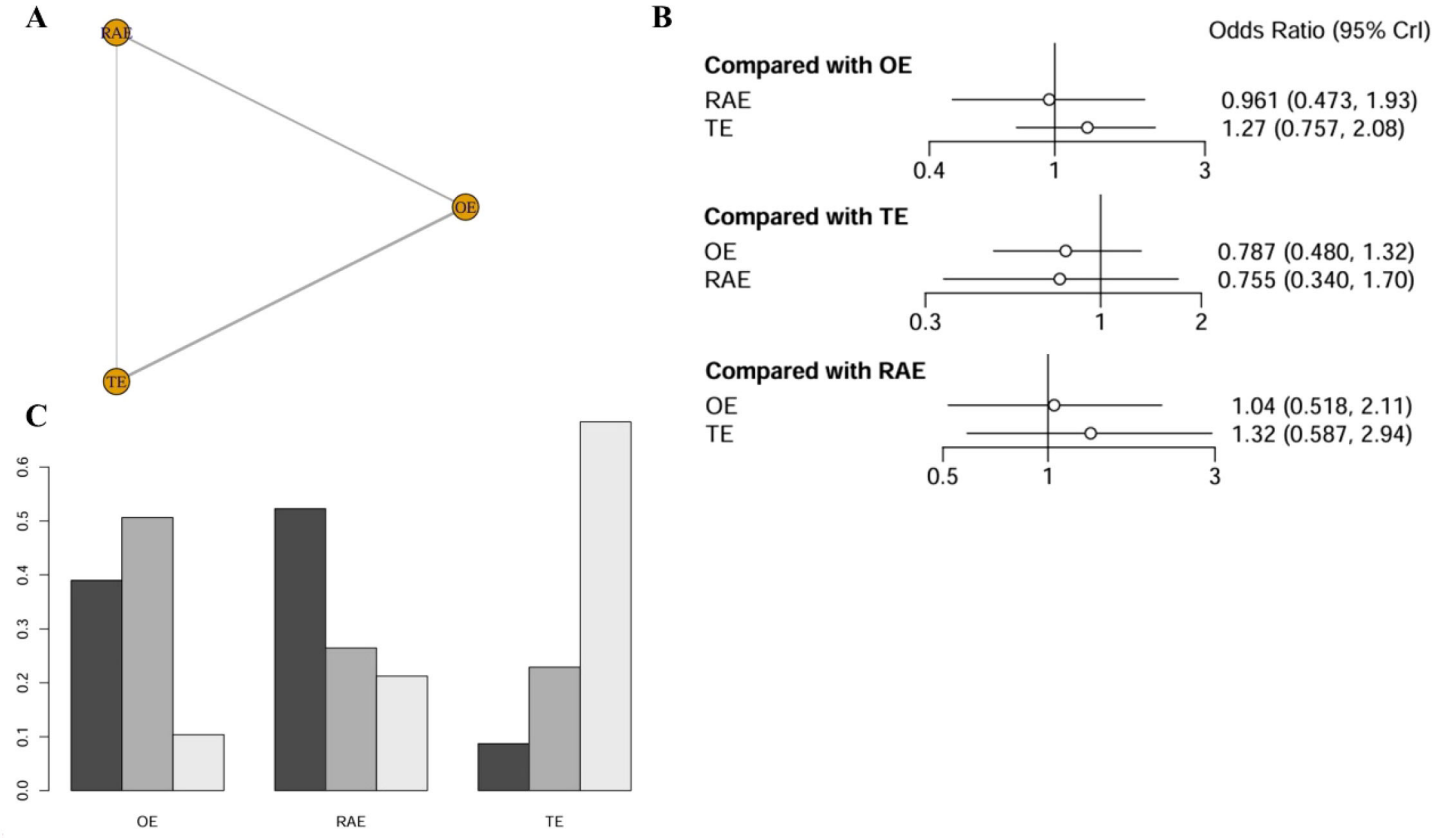
Network meta-analysis of overall complication rates. **(A)** The network evidence plot of overall complication rates; **(B)** The forest plot of overall complication rates; **(C)** The SUCRA probability ranking plot of overall complication rates.

The impact of various interventions on overall complication rates incorporated four studies comprising 645 patients, with network relationships depicted in **Figure 2A**. The analysis revealed no statistically significant differences in efficacy among the interventions (P > 0.05). Specifically, using OE as the reference group, the pooled odds ratios (ORs) with 95% CIs for RAMIE and VATE were 0.961 (0.473–1.93) and 1.27 (0.757–2.08), respectively. All confidence intervals included the null value of 1, indicating no statistically significant treatment effects (**Figure 2B**). Cumulative ranking probability analysis (SUCRA) indicated that RAMIE had the highest probability of reducing complications (SUCRA = 52.5%), followed by OE (38.4%) and VATE (9.1%). The ranking outcomes were illustrated in **Figure 2C**, with additional validation provided in the league table (refer to **Supplementary Table**).

#### Operative time

**Figure 3:**
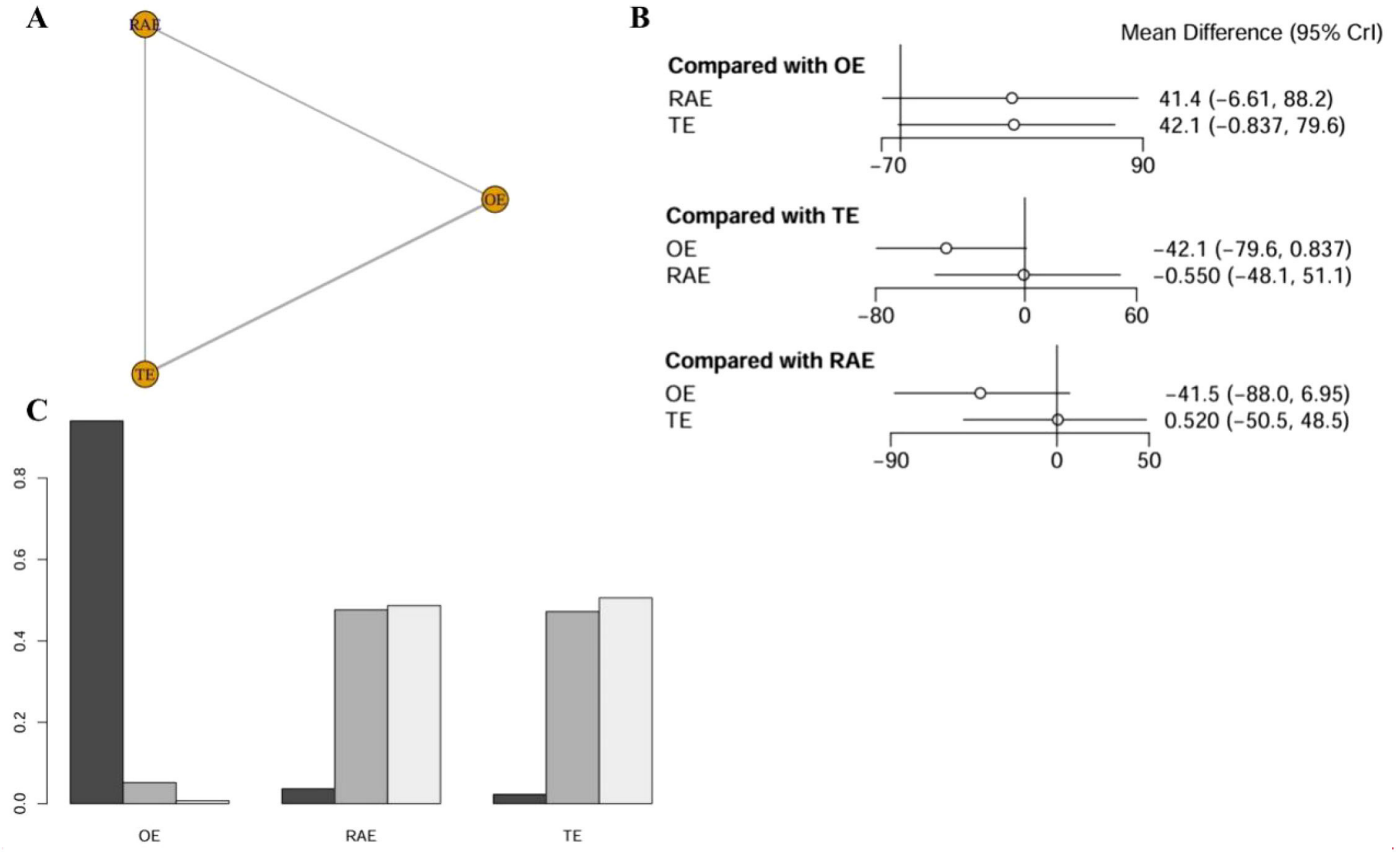
Network meta-analysis of operative time. **(A)** The network evidence plot of operative time; **(B)** The forest plot of operative time; **(C)** The SUCRA probability ranking plot of operative time.

The network meta-analysis assessing operative time incorporated data from five studies, encompassing 526 patients. The network diagram was depicted in **Figure 3A**. The findings revealed no statistically significant differences in operative time across the various surgical approaches, as indicated by all comparisons yielding P-values greater than 0.05. Using OE as the reference standard, the weighted mean differences (WMDs) for RAMIE and VATE were 41.4 (−6.61-88.2) and 42.1 (−0.837-79.6), respectively. Notably, all confidence intervals included the null value of 0 (**Figure 3B**). The SUCRA indicated that OE had the highest likelihood of reducing operative time, with a probability of 94.0%, significantly surpassing RAMIE at 3.7% and VATE at 2.3%. The ranking results were depicted in **Figure 3C** and league table (refer to **Supplementary Table**).

#### R0 resection rate

**Figure 4:**
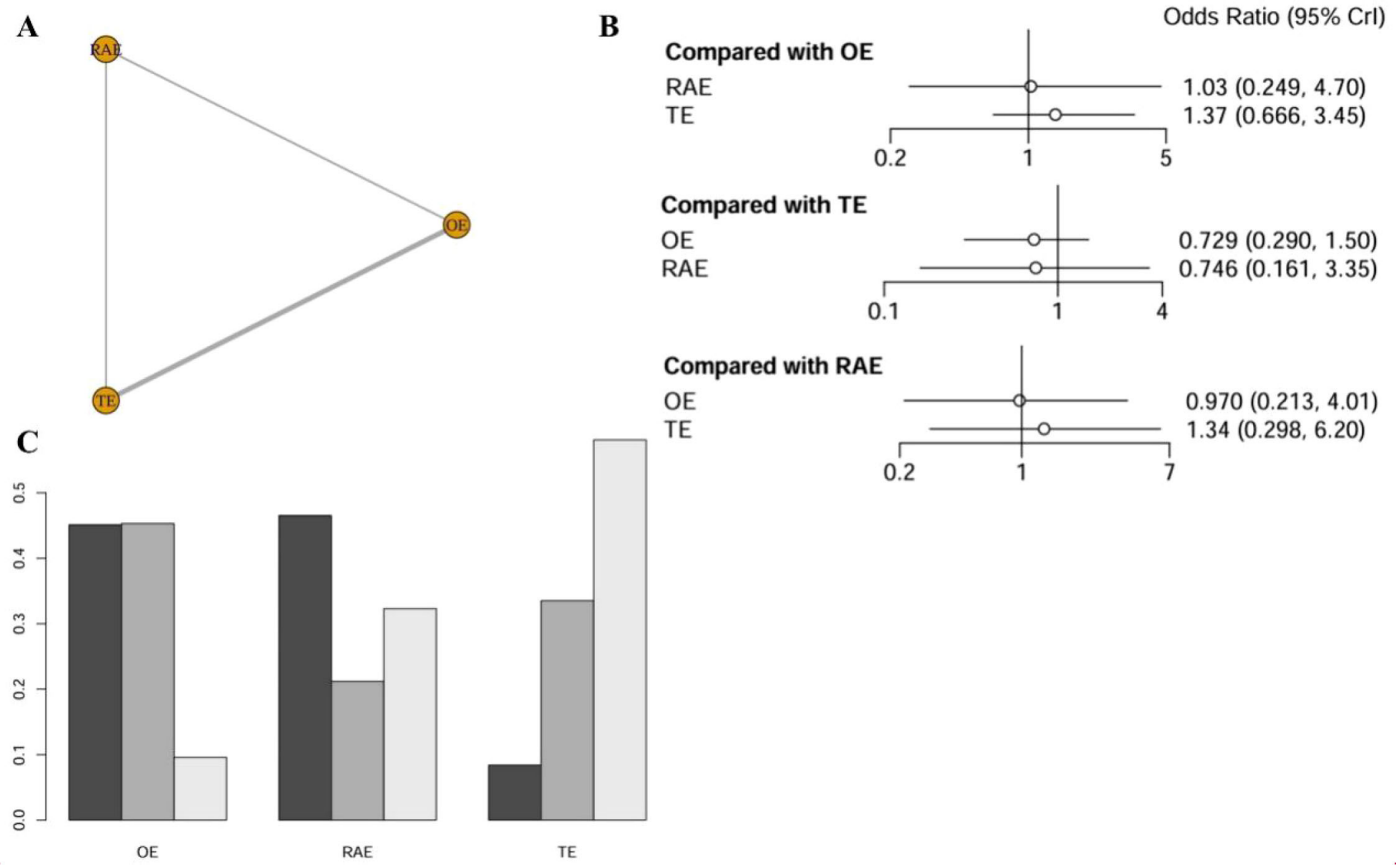
Network meta-analysis of R0 resection rate. **(A)** The network evidence plot of R0 resection rate; **(B)** The forest plot of R0 resection rate; **(C)** The SUCRA probability ranking plot of R0 resection rate.

The network meta-analysis of the R0 resection rate incorporated data from seven studies, encompassing 1,847 patients. The network diagram was depicted in **Figure 4A**. The analysis revealed no statistically significant differences in the effects of the various interventions. Specifically, when using OE as the reference point, the ORs for RAMIE and VATE were 1.03 (0.249-4.70) and 1.37 (0.666-3.45), respectively, as detailed in **Figure 4B**. According to the SUCRA presented in the ranking plot (**Figure 4C**), the hierarchy of effectiveness for achieving R0 resection was as follows: RAMIE (47.3%), OE (43.8%), and VATE (8.9%). Additional information was available in league table (refer to **Supplementary Table**).

#### Number of lymph nodes dissection

**Figure 5:**
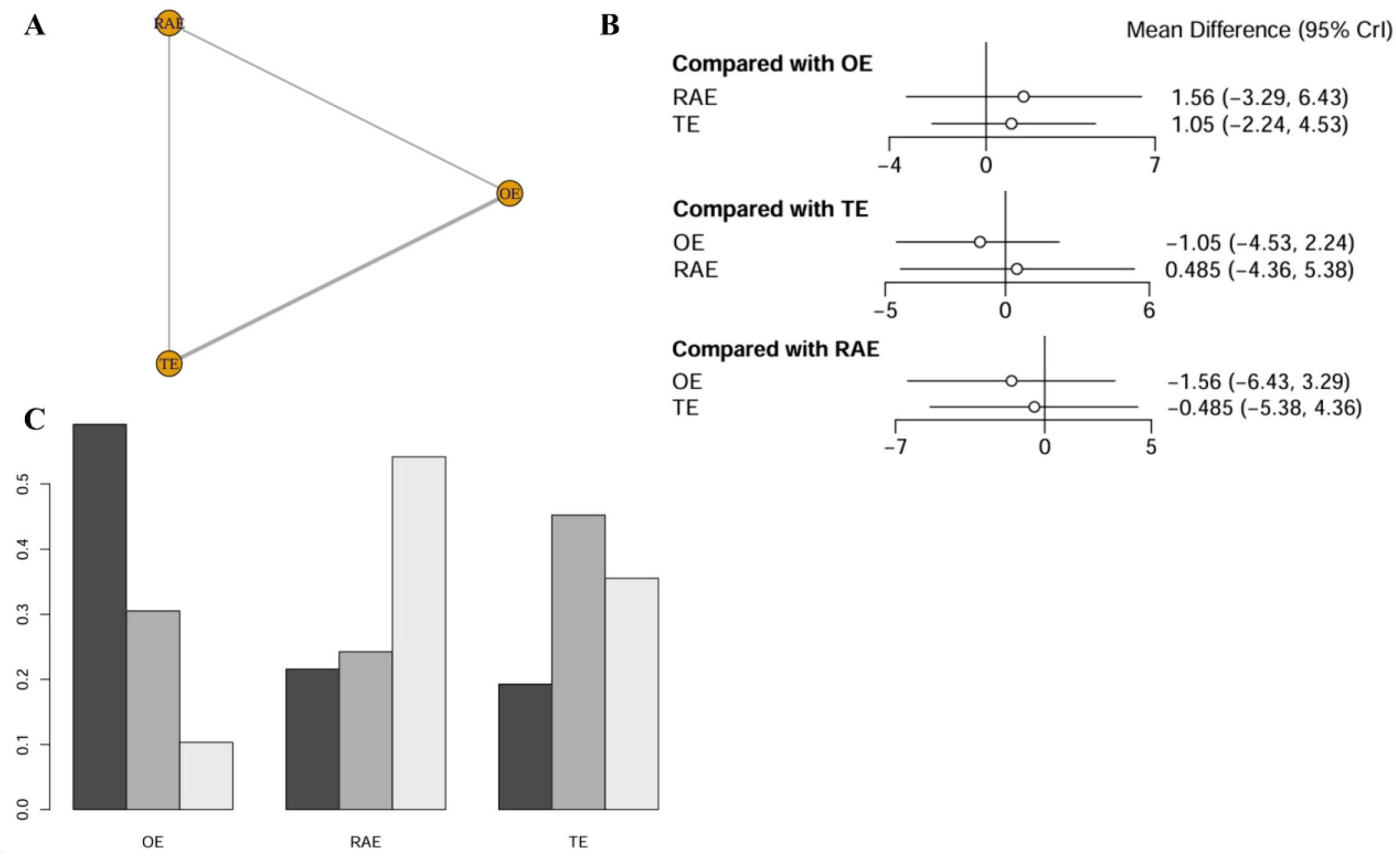
Network meta-analysis of number of lymph nodes dissection. **(A)** The network evidence plot of number of lymph nodes dissection; **(B)** The forest plot of number of lymph nodes dissection; **(C)** The SUCRA probability ranking plot of number of lymph nodes dissection.

The network meta-analysis assessing the number of lymph nodes dissection encompassed six studies with a total of 788 patients, systematically comparing the outcomes of various surgical techniques (**Figure 5A**). Statistical evaluation indicated no significant differences in number of lymph nodes dissected across the interventions (all comparisons P > 0.05). Specifically, when using OE as the reference, the WMDs for RAMIE and VATE were 1.56 (-3.29-6.43) and 1.05 (-2.24-4.53), respectively. Including the null value of 0 within all confidence intervals suggests the absence of significant treatment effects (**Figure 5B**). SUCRA indicated that OE had the highest probability of being superior in lymph node dissection (59.5%), markedly outperforming RAMIE (21.3%) and VATE (19.2%). The ranking results were illustrated in **Figure 5C** and league table (refer to **Supplementary Table**).

### 3-year overall survival

**Figure 6:**
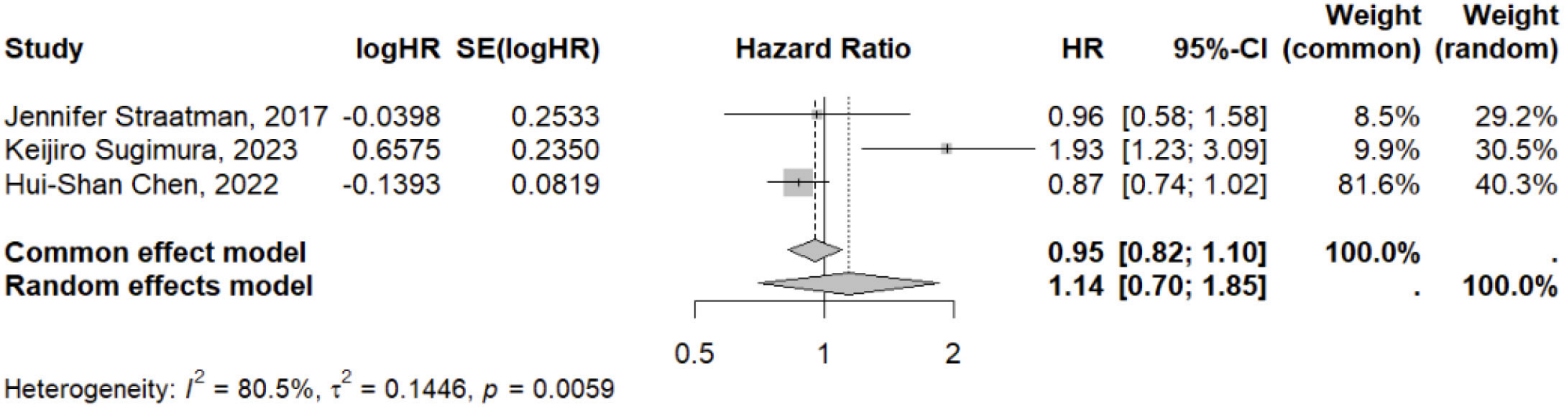
Network meta-analysis of 3-year OS. **(A)** The network evidence plot of 3-year OS; **(B)** The forest plot of 3-year OS; **(C)** The SUCRA probability ranking plot of 3-year OS.

The investigation of 3-year OS exclusively included studies focusing on OE and VATE interventions. Consequently, a meta-analysis was conducted solely on this outcome within the present study. The findings revealed that three studies, encompassing 1,436 patients, reported on 3-year OS. Using OE as the control group, the pooled effect size for VATE was 1.14 (0.70-1.85), indicating no statistically significant difference. For further details, refer to the accompanying **Figure 6**.

### Sensitive analysis

A sensitivity analysis excluding one RCT study was performed, and the results remained consistent with the primary analysis, demonstrating robustness in the findings.

### Operative time

**Figure 7:**
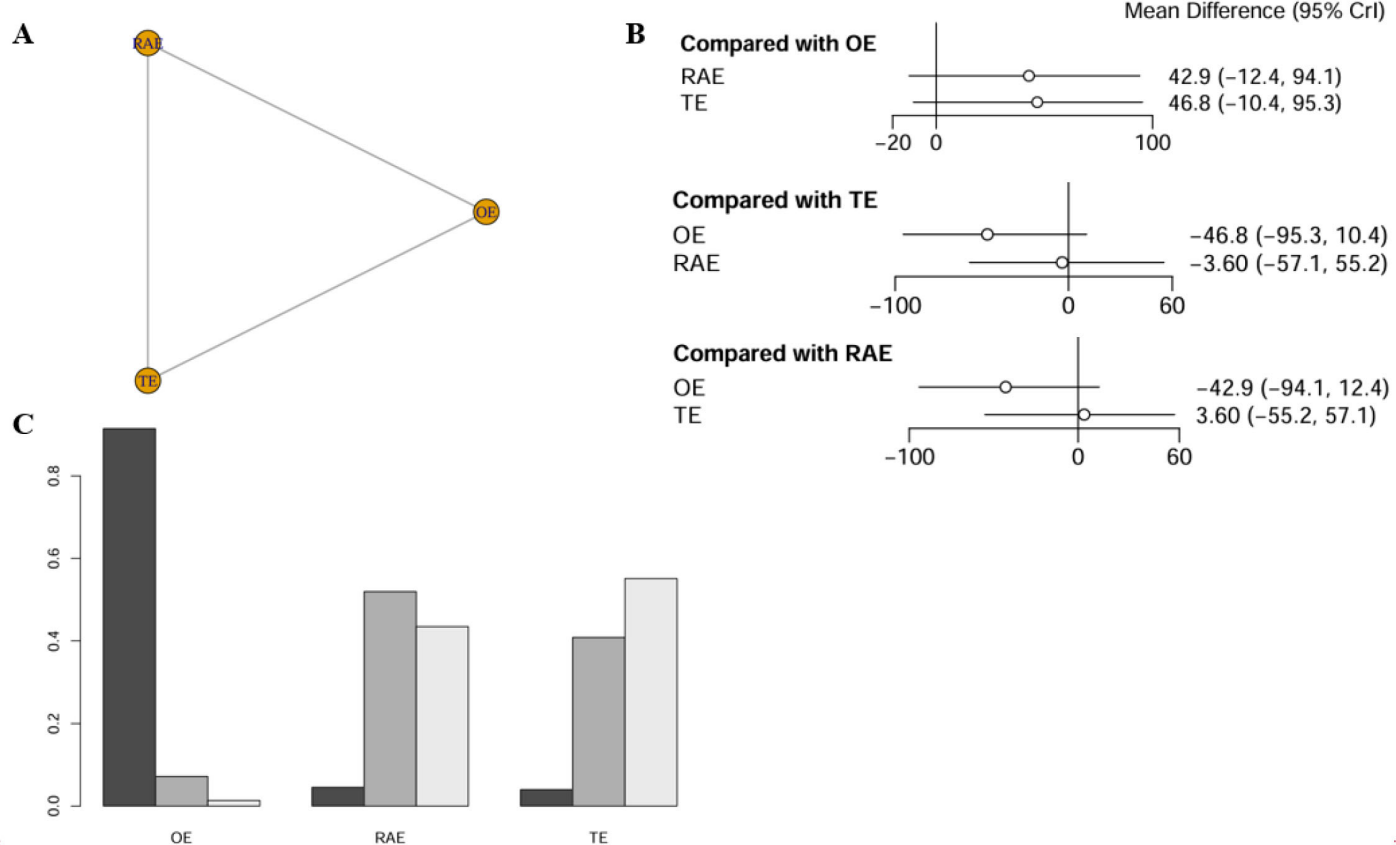
Sensitivity analysis of operative time.

The results were robust and no difference was found after excluding one RCT. The rankings were OE (94.0%), RAMIE (3.7%), VATE (2.3%) (**Figure 7**).

### R0 resection rate

**Figure 8:**
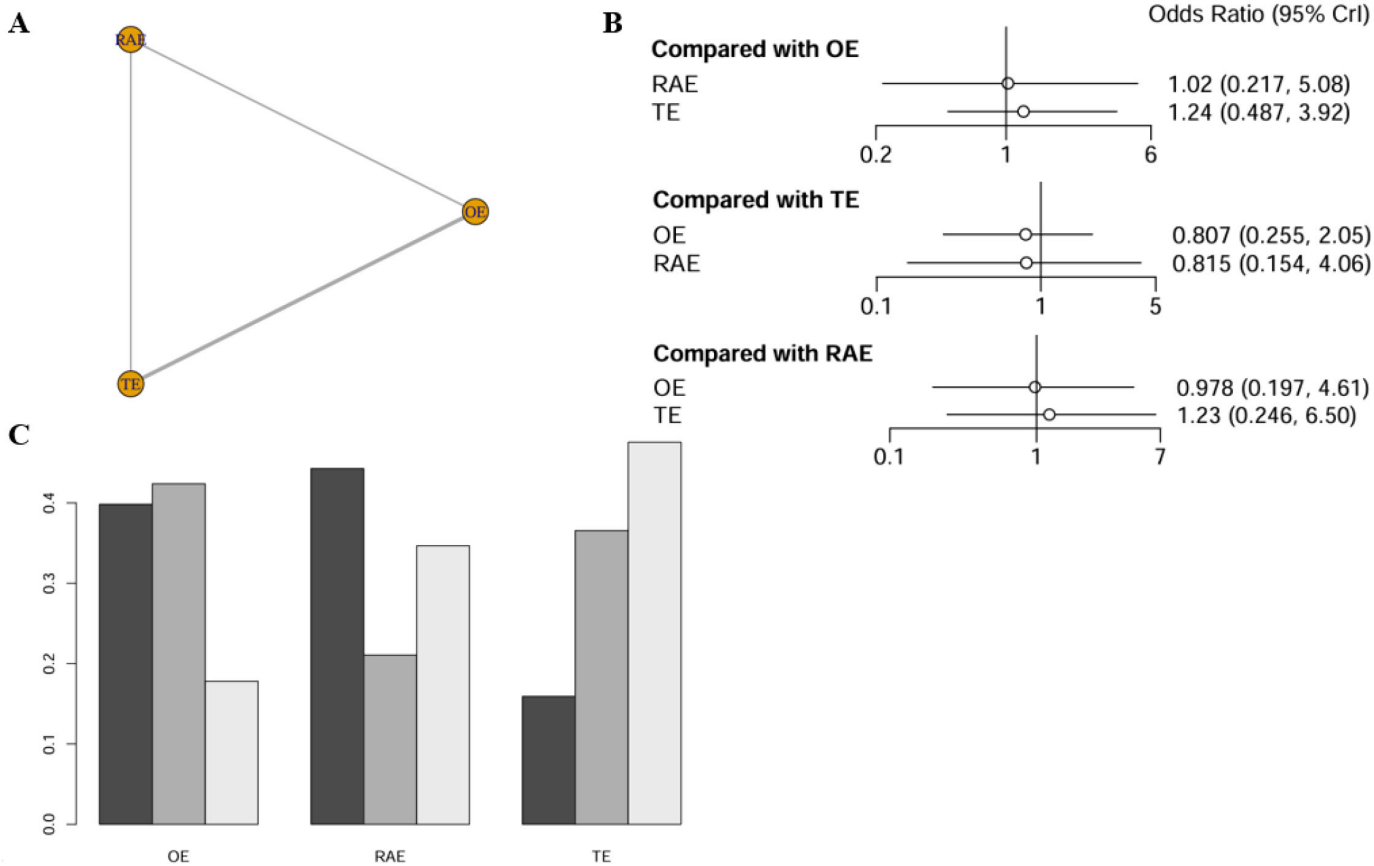
Sensitivity analysis of R0 resection rate.

Sensitivity analysis after excluding one RCT revealed no outcome changes, indicating robust findings. The rankings were RAMIE (44.3%), OE (39.8%), and VATE (15.9%) (**Figure 8**).

### Number of lymph nodes dissection

**Figure 9:**
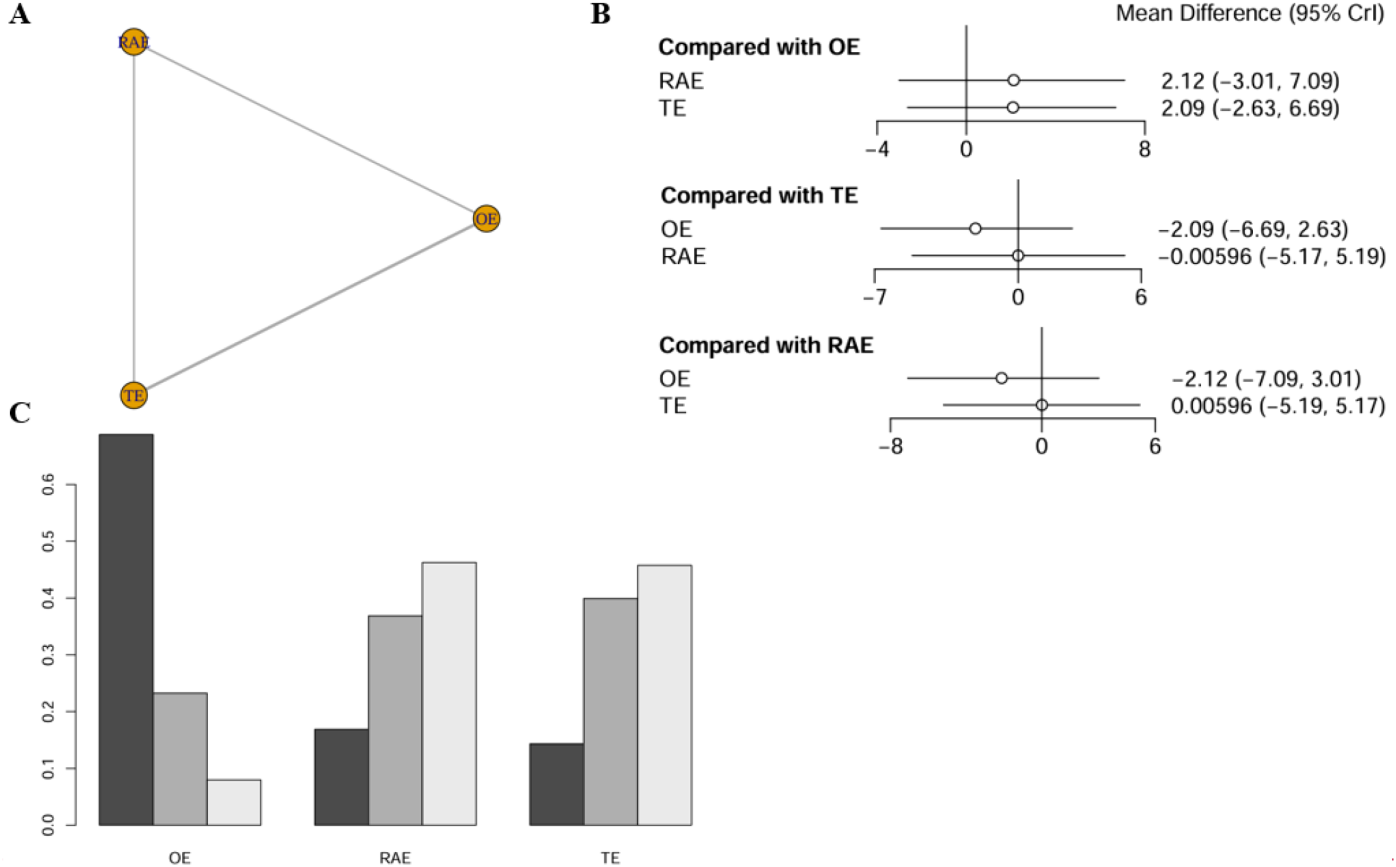
Sensitivity analysis of number of lymph nodes dissection.

Sensitivity analysis after excluding one RCT showed no changes in results, demonstrating robust findings. The ranking probabilities were OE (68.8%), RAMIE (16.9%), and VATE (14.3%) (**Figure 9**).

### 3-year OS

**Figure 10:**
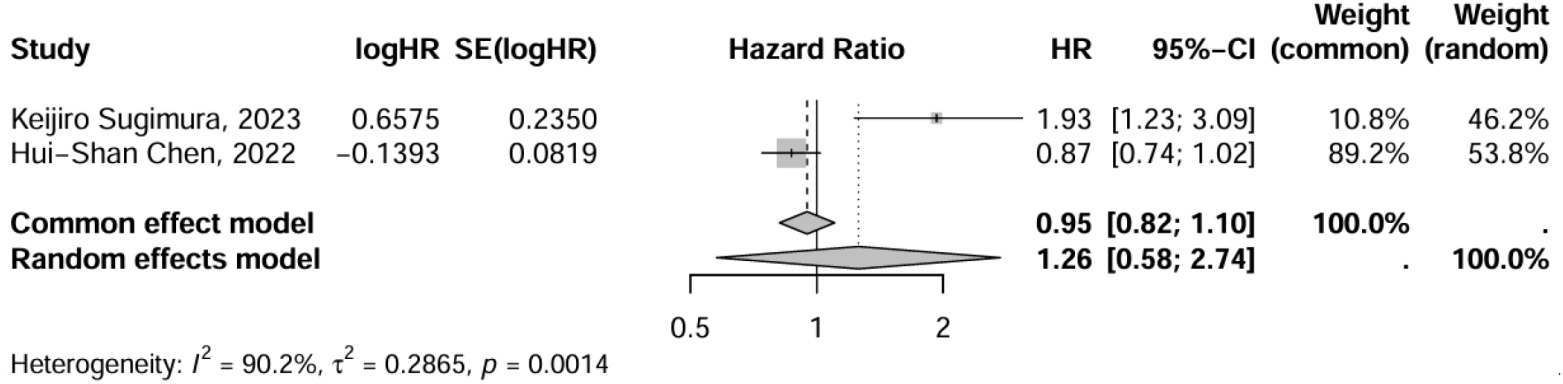
Sensitivity analysis of 3-year OS.

Sensitivity analysis after excluding one RCT showed no changes in outcomes, confirming the robustness of the results. Using OE as the reference group, the pooled effect estimate for VATE was 1.26 (95% CI: 0.58, 2.74) (**Figure 10**).

## Discussion

The combination of neoadjuvant therapy with surgical resection has now become the standard treatment modality for locally advanced EC. Over decades of continuous refinement and innovation, radical esophagectomy has undergone a remarkable transformation, evolving from conventional open procedures to minimally invasive approached. In recent years, robot-assisted surgery has become more and more popular, which reduces the trauma of patients and raises the requirements for surgeons. The neoadjuvant therapy-induced cytotoxic effects on the tumor may enhance the fragility of both the neoplastic and adjacent tissues, thereby elevating the susceptibility to rupture or hemorrhage during surgical intervention, this may augment the complexity and associated risks of the surgical procedure (25). In addition, neoadjuvant chemoradiotherapy may induce immunosuppression, which could compromise the body’s self-repair capacity by impairing the immune system’s critical role in tissue regeneration 27). Importantly, Immune checkpoint inhibitors (ICIs) have the potential to induce adverse effects, including cardiotoxicity, hepatotoxicity, and thyroid dysfunction, which are linked to endocrine disruptions. These complications may adversely affect postoperative recovery in patients with EC and elevate the risk of perioperative morbidity (26). The optimal surgical strategy for patients undergoing neoadjuvant therapy continues to be a topic of considerable discussion within the medical community. A systematic literature review indicates that contemporary clinical studies and meta-analyses have largely concentrated on pairwise comparisons. For example, a meta-analysis by Zhang et al. (27) thoroughly assessed the efficacy differences between RAMIE and VATE in the context of thoracic surgery. Their findings revealed that RAMIE offers notable technical advantages over VATE in certain anatomical areas, such as mediastinal and left recurrent laryngeal nerve lymph node dissection. Furthermore, the group undergoing RAE demonstrated a favorable trend in 3-year OS.

Substantial evidence gaps persist in direct comparative studies of RAMIE, VATE, and OE for EC following neoadjuvant therapy. This situation introduces a risk of bias in clinical decision-making due to reliance on multiple indirect comparisons. The analysis aimed to elucidate comparative advantages and limitations in performing radical esophagectomy following neoadjuvant therapy. This study represents the inaugural application of network meta-analysis methodology to synthesize both direct and indirect evidence through the development of a Bayesian probability model. This approach overcomes the constraints of traditional pairwise comparisons, enabling the concurrent quantitative evaluation and ranking of the three surgical procedures. Consequently, it offers a more comprehensive evidence-based foundation for optimizing the selection of surgical strategies.

This network meta-analysis systematically evaluated data from 7 randomized or observational studies involving 1,847 patients with EC who underwent neoadjuvant therapy. The study assessed three surgical approaches-OE, RAMIE, and VATE-focusing on critical clinical outcomes, including postoperative complication rates, operative time, R0 resection rate, and 3-year OS.

The network meta-analysis demonstrated no statistically significant differences in operative time, R0 resection rate, lymph node yield, or overall complications among OE, RAMIE, and VATE. However, intervention rankings revealed clinically relevant gradients: OE exhibited superior probability for reducing operative time (OE > RAMIE > VATE) and optimizing lymphadenectomy (OE > RAMIE >VATE), while RAMIE ranked highest for complication minimization (RAMIE > OE > VATE).For 3-year OS, pairwise comparisons between OE and VATE showed equivalent survival outcomes (HR = 1.12, 95% CI = 0.87-1.44), with sensitivity analyses confirming robustness despite predominantly observational evidence. These results suggest that while statistical equivalence exists across surgical modalities, nuanced performance differences may guide procedure selection based on individualized clinical priorities.

Esophagectomy for EC is a complex surgical procedure characterized by a relatively high incidence of complications, encompassing esophageal dissection, lymphadenectomy, and esophagogastric anastomosis. Common postoperative complications include pulmonary infections, hoarseness resulting from recurrent laryngeal nerve injury, anastomotic leakage, and chylothorax (28). The advent and increasing utilization of RAMIE have demonstrated substantial benefits in mitigating postoperative complications. Notably, RAMIE showed significant decrease in cardiopulmonary complications. Compared to traditional OE, RAMIE has decreased incidence of postoperative pneumonia (29). OE may offer advantages in terms of operation time, because OE can be more direct and effective, particularly with complex tumors. With advancements in surgeon skill and procedural optimization, the operative time for both VATE and RAMIE has shortened significantly. While initial studies noted longer durations for RAMIE—attributed to the learning curve associated with 3D visualization, robotic instrumentation, and team integration—increasing surgical experience leads to greater efficiency (30). As surgeons and operating room teams climb the learning curve, streamlined workflows and improved intraoperative management allow RAMIE to achieve operative times comparable to, or even shorter than, those of conventional minimally invasive esophagectomy (31). Furthermore, RAMIE demonstrates superior outcomes in recurrent laryngeal nerve preservation, a critical factor for reducing postoperative voice and swallowing dysfunction, thereby significantly enhancing patients’ quality of life (32). Nevertheless, OE is associated with greater invasiveness and is linked to increased intraoperative blood loss, postoperative complications, and postoperative pain when compared to RAMIE or VATE (33).

The network meta-analysis of included studies revealed no statistically significant differences in R0 resection rates among the three surgical approaches. Although cumulative ranking probabilities indicated RAMIE had the highest likelihood of achieving R0 resection (SUCRA = 47.3%), the minimal disparity compared with OE (SUCRA = 43.8%) and wide confidence intervals collectively suggest clinical equipoise across these techniques. This finding underscores that while technical variations exist in operative details and postoperative recovery profiles, comparable oncological efficacy in R0 resection can be attained with RAMIE, OE, and VATE under specific clinical contexts. These results provide clinicians with greater flexibility in procedure selection, supported by existing evidence demonstrating therapeutic equivalence in tumor eradication outcomes among surgical modalities. A meta-analysis encompassing several studies revealed no statistically significant difference between RAMIE and OE concerning the critical prognostic indicator of the R0 resection rate (34). A prospective cohort study conducted by Tokyo Medical University, encompassing more than ten years of postoperative follow-up, established clinical equivalence in R0 resection rates and long-term survival outcomes between the RAMIE and VATE groups. Additionally, the study highlighted the superior efficacy of RAMIE in minimizing procedure-related complications and intraoperative blood loss (35). Given the high risk associated with esophagectomy, it is may better to centralize patients in high-volume hospitals (36).

The quantity of excised lymph nodes serves as a crucial parameter in assessing surgical efficacy in treating EC. This study demonstrated no statistically significant differences in the total number of dissected lymph nodes among the OE, VATE, and RAMIE groups (P > 0.05). However, network meta-analysis revealed that OE exhibited superior performance in cumulative ranking of lymph node dissection quantity (SUCRA = 59.5%), demonstrating significantly higher values compared to both RAMIE (SUCRA = 21.3%) and VATE (SUCRA = 19.2%). The three surgical approaches (OE, RAMIE, and VATE) demonstrate distinct characteristics and advantages in lymph node dissection. Compared to minimally invasive approaches, open esophagectomy (OE) offers an inherent advantage for lymph node dissection due to its larger operative space and more straightforward manual access. In contrast, VATE, as a minimally invasive approach, offers clinical advantages including reduced operative trauma and expedited postoperative recovery. The study by Li Zhenhua et al. (37) demonstrated comparable lymph node yield between VATE and OE, highlighting the technically demanding nature of VATE requiring advanced surgical proficiency. Notably, the procedure’s inherent limitations of two-dimensional visualization may potentially compromise dissection completeness in anatomically complex cases, particularly when operating surgeons lack sufficient thoracoscopic expertise. In recent years, RAMIE has attracted increasing attention in EC surgery. The robotic system’s wristed instruments, like the EndoWrist in the da Vinci platform, offer multiple degrees of freedom, surpassing human hand dexterity in tight surgical spaces. Tremor filtration removes hand tremors, boosting the surgeon’s ability to work in complex areas. These features enhance the precision and flexibility of robotic-assisted surgery., In addition, the RAMIE offers greater comfort to the first operator that should not be underestimated (38). Lei J et al. (39) showed although RAMIE has no significant superiority in total lymph node yield, but stratified analyses of nodal stations in existing clinical investigations have identified enhanced dissection efficacy in anatomically critical zones, particularly the left recurrent laryngeal nerve basin and mediastinal regions where conventional approaches encounter technical constraints. A meta-analysis comparing the short-term outcomes between RAMIE and conventional minimally invasive esophagectomy found that RAMIE offers advantages in lymph node dissection, particularly yielding a higher number of retrieved lymph nodes in the left recurrent laryngeal nerve area (40). Furthermore, the first Irish study on robotic esophagectomy also demonstrated that robotic surgery achieved a greater lymph node harvest compared to other surgical approaches (41). Therefore, we conclude that with the growing proficiency of surgeons in robotic techniques, RAMIE demonstrates a superior ability for lymph node dissection compared to OE or VATE, particularly in challenging areas such as the left recurrent laryngeal nerve region.

Long-term survival continues to be the primary endpoint in oncological research. The current study demonstrated similar 3-year OS rates between the OE and VATE cohorts, with 64.5% and 67.2% rates. The analysis revealed no statistically significant difference in survival outcomes (P > 0.05). These results indicate that with ongoing advancements in thoracoscopic surgical techniques, VATE has achieved long-term oncological outcomes comparable to the traditional open approach, underscoring its clinical non-inferiority in the comprehensive management of cancer. This observation has been consistently confirmed by multiple high-quality studies in recent years. Specifically, findings from the Japanese 10-year clinical trial JCOG1409, presented at the 2024 American Society of Clinical Oncology (ASCO) Annual Meeting, demonstrated comparable 3-year OS rates between the VATE and OE groups (82.0%, 70.9%, respectively; P > 0.05). These data indicate comparable long-term survival outcomes between the two surgical approaches (39). However, the equivalence of surgical approaches regarding oncological efficacy and long-term prognosis remains debated. A Japanese retrospective study from the Osaka International Cancer Institute reported divergent conclusions. Their analysis demonstrated significantly superior 3-year disease-free survival (DFS: 71.4%, 50.5%; P = 0.004) and overall survival (OS: 80.3%, 61.2%; P = 0.002) rates in the VATE group compared to the OE cohort (40). Current evidence indicates that VATE has overcome technical bottlenecks, and its long-term survival outcomes are comparable to those of OE. This is mainly attributed to developing of high-definition imaging systems and establishing of standardized lymph node dissection procedures. However, the heterogeneity of results from different studies suggests that the potential advantages of VATE may be influenced by multiple factors such as the surgical team’s experience, case selection criteria, and perioperative management strategies. Notably, although RAE does not show significant survival benefits, its precise operation characteristics may offer new solutions for dissection high-risk anatomical regions.

### Limitations

While the statistical difference was insignificant, the observed ranking gradient may indicate a clinical trend among various surgical techniques for EC in practical settings. Factors such as the limited sample size of the included studies and substantial heterogeneity-stemming from variations in surgical procedures across different centers-might obscure the true differences. Without statistical significance, clinical gradients can serve as an additional reference for informing clinical decision-making.

### Conclusions

The network meta-analysis indicated no significant differences in key oncological outcomes, such as survival and R0 resection rates, among the three esophagectomy approaches following neoadjuvant therapy, demonstrating overall therapeutic equivalence. Nonetheless, distinct perioperative profiles were observed. RAMIE was associated with a reduced risk of complications, whereas OE exhibited shorter operative durations compared to minimally invasive techniques. These findings suggest that clinical decision-making should be individualized, taking into account tumor-specific objectives, patient comorbidities, and the availability of institutional resources. For example, OE may be prioritized in situations requiring expedited procedures, while RAMIE may be preferred in cases with anticipated involvement of the left recurrent laryngeal nerve lymph nodes, necessitating meticulous dissection to minimize postoperative morbidity. Future research should concentrate on large-scale prospective studies with standardized protocols to validate these trends, while explore technical refinements and precision stratification based on anatomical risk profiles.

## Data Availability

The original contributions presented in the study are included in the article/**Supplementary Material**. Further inquiries can be directed to the corresponding author.
