# Supplementary material for: Robot-assisted minimally invasive esophagectomy versus video-assisted thoracoscopic esophagectomy versus open esophagectomy for locally advanced esophageal cancer after neoadjuvant therapy: a systematic review and network meta-analysis
Source: Front Oncol. 2025 Nov 20;15:1631672. doi: 10.3389/fonc.2025.1631672 (PMC12675205; doi:10.3389/fonc.2025.1631672)

**Two-tailed contingency table for pairwise comparison of overall incidence of comorbidities**

|  | RAMIE | OE | VATE |
| --- | --- | --- | --- |
| RAMIE | - | 0.025 (-0.676,0.728) | 0.258 (-0.543,1.041) |
| OE | -0.025 (-0.728, 0.676) | - | 0.232 (-0.282,0.732) |
| VATE | -0.258 (-1.041, 0.543) | -0.232 (-0.732, 0.282) | - |

**Overall incidence rate of comorbidities local consistency test, node analysis**


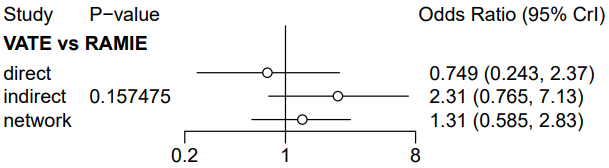


**Heterogeneity test for the overall incidence of comorbidities**


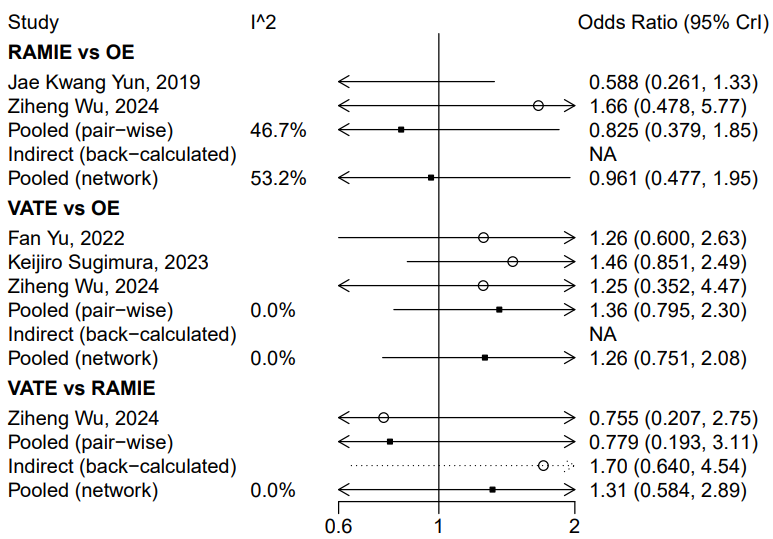


**Pairwise comparison table of operation time**

|  | RAMIE | OE | VATE |
| --- | --- | --- | --- |
| RAMIE | - | -41.350 (-87.580, 7.163) | 0.757 (-50.650, 49.190) |
| OE | 41.350 (-7.163, 87.580) | - | 42.070 (-1.067, 79.350) |
| VATE | -0.757 (-49.190, 50.650) | -42.07 (-79.35, 1.067) | - |

**Local consistency test of operation time, node analysis**


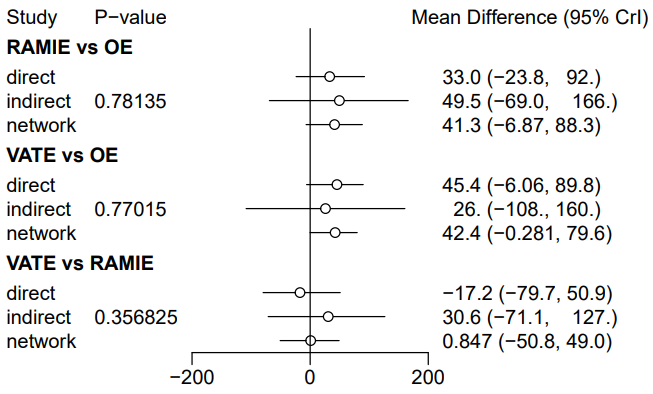


**Test for heterogeneity of operation time**


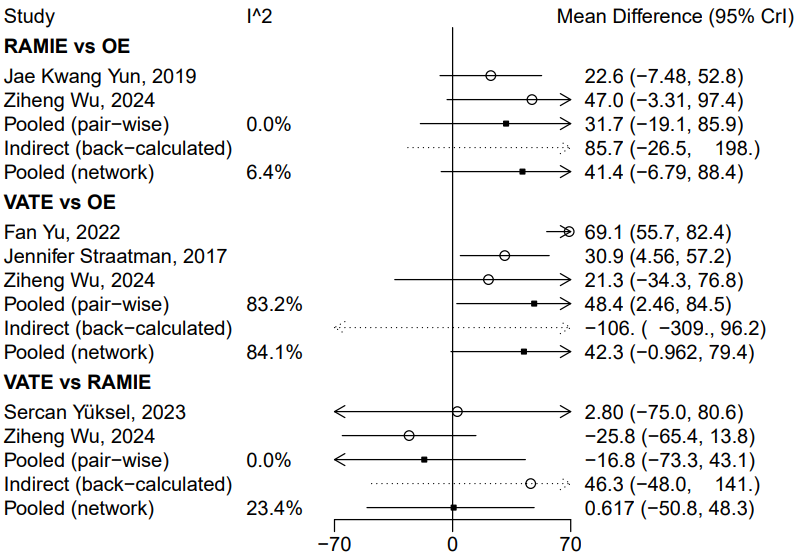


**Pairwise comparison of R0 resection rates using contingency table**

|  | RAMIE | OE | VATE |
| --- | --- | --- | --- |
| RAMIE | - | -0.057 (-1.590, 1.390) | 0.269 (-1.261, 1.791) |
| OE | 0.057 (-1.390,1.590) | - | 0.309 (-0.418, 1.238) |
| VATE | -0.269 (-1.791, 1.261) | -0.309 (-1.238, 0.418) | - |

**Local consistency test of R0 resection rate, node analysis**


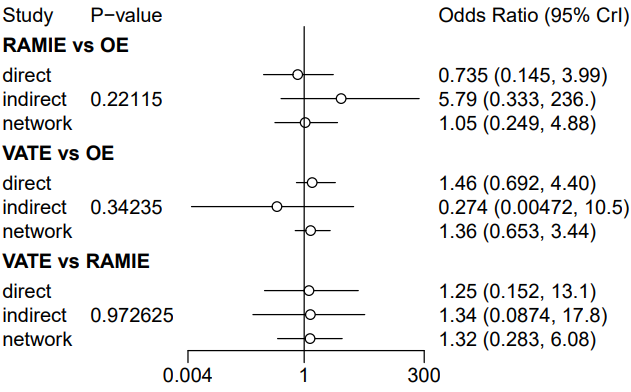


**Heterogeneity test of R0 resection rate**


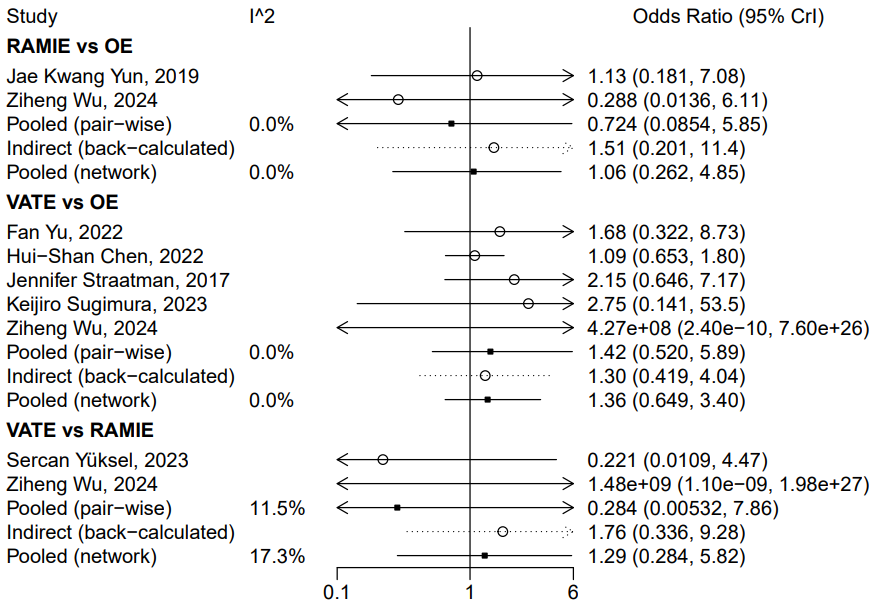


**Contingency table comparing the number of lymph node dissections in pairs**

|  | RAMIE | OE | VATE |
| --- | --- | --- | --- |
| RAMIE | - | -1.630 (-6.366, 3.103) | -0.560 (-5.461, 4.390) |
| OE | 1.630 (-3.103, 6.366) | - | 1.069 (-2.328, 4.537) |
| VATE | 0.560 (-4.390, 5.461) | -1.069 (-4.537, 2.328) | - |

**Local consistency test of the number of lymph node dissections, node analysis**


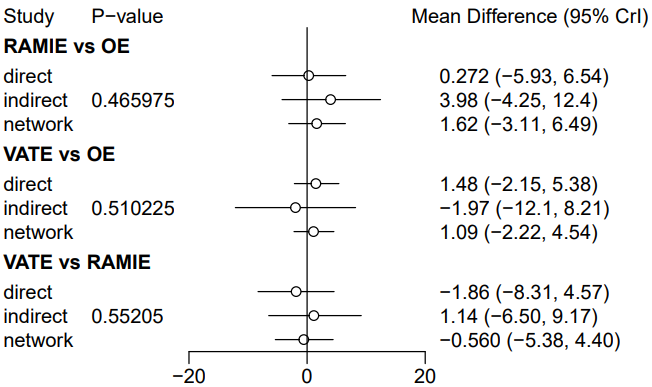


**Heterogeneity test of the number of lymph node dissections**


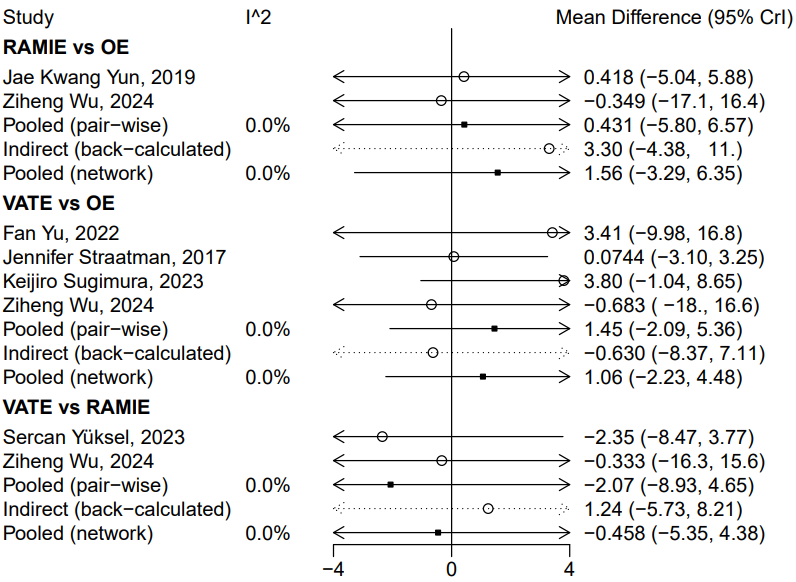

Supplement: Supplementary file 1 [file Table1.docx]
